# Real-time imaging of glutamate transients in the extracellular space of acute human brain slices using a single-wavelength glutamate fluorescence nanosensor

**DOI:** 10.1038/s41598-022-07940-8

**Published:** 2022-03-10

**Authors:** Sebastian Brandner, Simon Aicher, Sarah Schroeter, Izabela Swierzy, Thomas M. Kinfe, Michael Buchfelder, Anna Maslarova, Andreas Stadlbauer

**Affiliations:** 1 Department of Neurosurgery, University Hospital Erlangen, Schwabachanlage 6, 91054 Erlangen, Germany; 2 Division of Functional Neurosurgery and Stereotaxy, University Hospital Erlangen, Erlangen, Germany; 3 Institute of Medical Radiology, University Clinic St. Pölten, Karl Landsteiner University of Health Sciences, St. Pölten, Austria; 4 Center for Musculoskeletal Surgery Osnabrück (OZMC), Klinikum Osnabrück, Osnabrück, Germany

**Keywords:** Neuronal physiology, Synaptic transmission

## Abstract

Glutamate is the most important excitatory neurotransmitter in the brain. The ability to assess glutamate release and re-uptake with high spatial and temporal resolution is crucial to understand the involvement of this primary excitatory neurotransmitter in both normal brain function and different neurological disorders. Real-time imaging of glutamate transients by fluorescent nanosensors has been accomplished in rat brain slices. We performed for the first time single-wavelength glutamate nanosensor imaging in human cortical brain slices obtained from patients who underwent epilepsy surgery. The glutamate fluorescence nanosensor signals of the electrically stimulated human cortical brain slices showed steep intensity increase followed by an exponential decrease. The spatial distribution and the time course of the signal were in good agreement with the position of the stimulation electrode and the dynamics of the electrical stimulation, respectively. Pharmacological manipulation of glutamate release and reuptake was associated with corresponding changes in the glutamate fluorescence nanosensor signals. We demonstrated that the recently developed fluorescent nanosensors for glutamate allow to detect neuronal activity in acute human cortical brain slices with high spatiotemporal precision. Future application to tissue samples from different pathologies may provide new insights into pathophysiology without the limitations of an animal model.

## Introduction

Glutamate serves as the major neurotransmitter in the mammalian brain, mediating 90% of the excitatory neurotransmission^1^. Glutamatergic synapses are essentially involved in the physiological and neurobiological mechanisms of all forms of behavior including perception and consciousness. Variations in the strength of connectivity at glutamatergic synapses are considered to be the cellular mechanisms underlying memory and learning^2^. Furthermore, glutamate-mediated neurotransmission is also thought to play a key role in several neurological disorders, such as neurodegenerative disorders and epilepsy^3,4^, and dysregulation of glutamate is implicated in receptor-mediated excitotoxicity following stroke and traumatic brain injury^5^.

A very simplified view is that glutamate is released into the synaptic cleft when action potentials depolarize the presynaptic membrane^6^ and activates postsynaptic glutamate receptors^7^ to trigger current flow in postsynaptic neurons. Remaining glutamate is rapidly removed from the synaptic cleft via absorption by astrocytes, a phenomenon known as glutamate clearance^8^, in order to terminate the glutamate signal^9,10^. However, it has also been shown that glutamate escapes from the synaptic cleft, generating extrasynaptic glutamate dynamics and activating extrasynaptic receptors (glutamate spillover effect)^11,12^ that regulate a variety of important neural and glial functions^13^. Consequently, assessment of the temporal and spatial dynamics of both glutamate release and clearance is crucial for understanding the physiology and pathophysiology of excitatory neurotransmission.

Established methods to study extracellular glutamate transients are microdialysis^14,15^, enzyme-linked fluorescence assays^16,17^, or enzymatic glutamate-selective microelectrodes^18,19^. However, these tools provide poor signal-to-noise ratio and targetability combined with only limited spatial and/or temporal resolution. Considering the prominent role of glutamate in excitatory neurotransmission and the importance for accurate and precise quantification of rapid glutamate transients in living tissue, tools that are capable of detecting real-time changes in rapid local glutamate concentration changes are needed.

Optical detection of glutamate using genetically encoded fluorescent nanosensors composed of glutamate-binding proteins coupled to fluorescent proteins enhance the temporal and spatial resolution and offer the potential to investigate glutamate transients. During glutamate binding, the recognition domain undergoes a conformation change that perturbs the photophysical properties of the fluorophores, providing a readout for the recognition event and generating precise spatial and temporal information. Förster resonance energy transfer (FRET)-based nanosensors represent the most frequent form of genetically encoded fluorescent nanosensors and use a form of nonradiative energy transfer that occurs between two fluorophores with overlapping absorption and emission spectra in close proximity^20^. However, FRET-based glutamate nanosensors such as FLIPE (fluorescent indicator protein for glutamate)^21^ or GluSnFR (glutamate-sensitive fluorescent reporters)^22^ have relatively low sensitivity, since glutamate binding causes only a small conformational change in the recognition domain and therefore only a slight fluorescence efficiency change ^21,22^. A breakthrough in visualizing glutamate release in intact tissue was achieved with single-wavelength glutamate nanosensors such as iGlu_u_ (intensity-based GluSnFR ultrafast)^23^ that relay an optical signal via changes in a single fluorophore. This single fluorophore-based nanosensor, which was specially developed for accurate tracking of synaptic glutamate dynamics during high-frequency transmission, has a greater sensitivity and reacts faster, which is particularly essential for investigation of neurotransmitter dynamics. Upon glutamate binding, the circularly permuted enhanced green fluorescent protein (cpEGFP) β-barrel of the iGlu_u_ nanosensor is pulled together resulting in a fivefold increase in fluorescence^23^.

Both FRET- and single fluorophore-based nanosensors are almost exclusively used for cytosolic investigations providing no direct insight into the spatial and temporal dynamics of neurotransmitter, ion, and metabolite changes in the extracellular space (ECS). Dulla et al.^24^ developed a novel approach to investigate glutamate dynamics in the ECS, using a purified FRET-based sensor protein for glutamate loaded on acute rodent brain slices. Glutamate dynamics were investigated under several pharmacologic conditions during electrical stimulation^25^. However, glutamate dynamics has so far not been investigated in the human brain.

Resection specimens from epilepsy surgery provide a unique opportunity to study neuronal activity in-vitro in the human brain. In the past, such specimens used for the investigation of epileptiform activity or cortical spreading depolarizations have demonstrated increased resistance of the human slices to stimuli that easily induced network phenomena in animal models, and considerable modifications of ionic concentrations as well as disinhibition were necessary^26,27^. We were therefore interested to test whether glutamate fluorescent imaging can be performed in human specimens with a reasonable temporal resolution to study glutamate dynamics.

In the present study, we demonstrate for the first time the feasibility of glutamate biosensor imaging in acute human cortical brain slices obtained from patients who underwent epilepsy surgery using the ultrafast single-wavelength sensor iGlu_u_. The described method allows the unique possibility of studying human glutamate dynamics and its role in epilepsy as well as in other pathologies, subject to neurosurgical resection.

## Results

### Detection of stimulated glutamate transients in human cortical brain slices.

**Figure 1:**
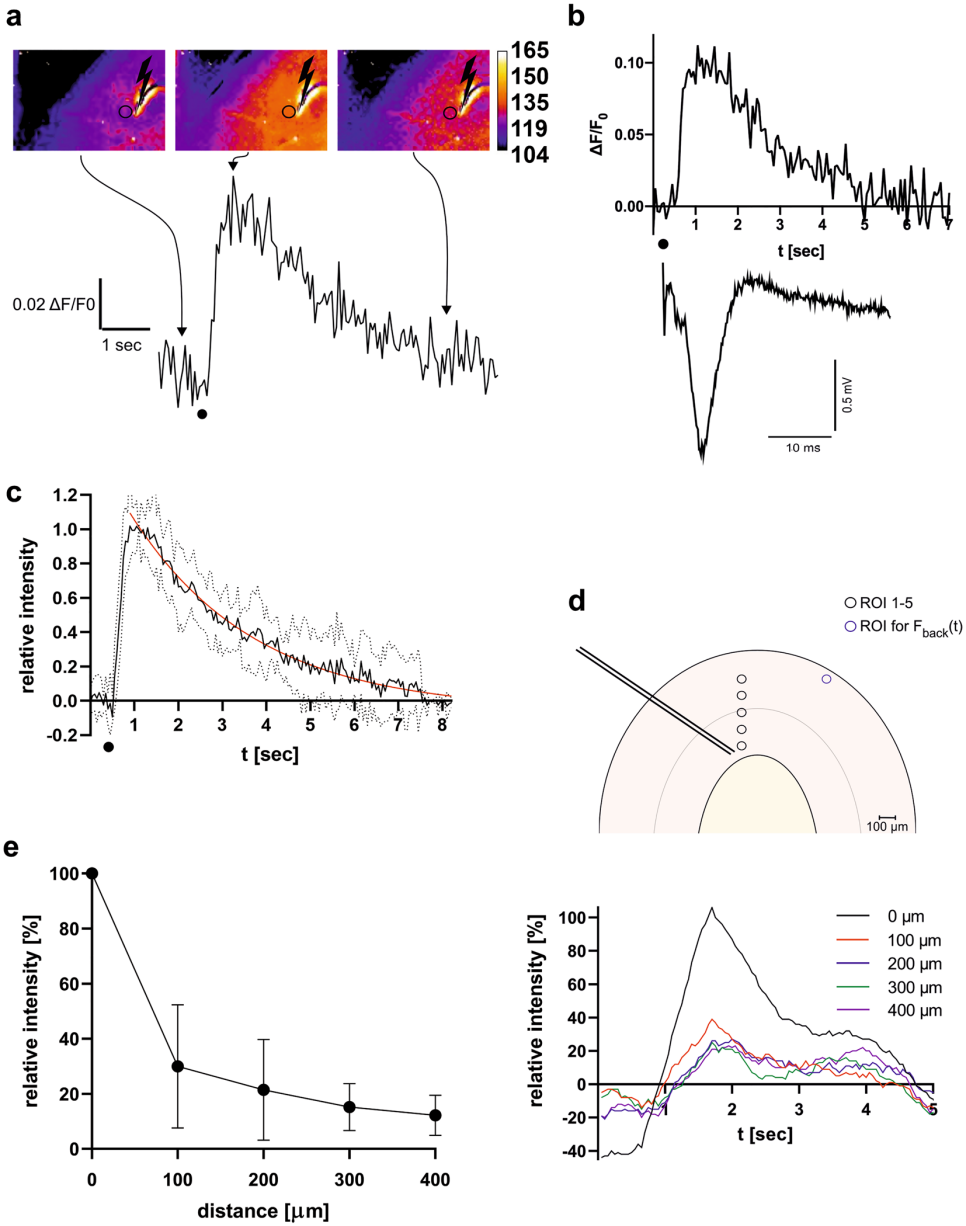
Characterization of the iGlu_u_ response to electrical stimulation in the extracellular space (ECS) of human cortical brain slices. **(a)** Series of false-color images of fluorescence microscopy images that show (from left to right) iGlu_u_ fluorescence prior to the electrical stimulation, at the peak intensity, and after exponential decay presumably due to glutamate clearance from the ECS. Color code is shown on the right. The time course of the iGlu_u_ fluorescence signal intensity for the experiment is depicted below. The time point of electrical stimulation is marked by a dot. **(b)** Example of glutamate nanosensor fluorescence intensity change (upper trace) and an extracellular field potential recording in layer II/III of the same slice (lower trace) upon a 0.1 ms bipolar stimulus (black dot) in cortical layer IV. Note the difference in temporal dynamics of the two signals: seconds for glutamate transients, milliseconds for field potentials. **(c)** Temporal characterization of the iGlu_u_ fluorescence intensity change. Black line—averaged iGlu_u_ fluorescence signal course from 63 stimulation courses from 7 human brain slices of 7 different patients; red line—exponential fitting curve; dotted lines—standard deviation. **(d)** Spatial characterization of the iGlu_u_ fluorescence intensity change. The fluorescence microscopy signal courses were measured at different regions of interest (ROIs) starting at the direct proximity (within 20 µm) of the stimulation electrode and with increasing distance from the stimulation electrode in 100 µm steps (upper image). The lower graph shows the different fluorescence levels of these ROIs following the same electrical stimulus. With increasing distance to the stimulation electrode, a decrease in maximum fluorescence level can be seen. **(e)** Spatial dependence of the peak signal intensity of the iGluu fluorescence intensity change (n = 8 slices, 7 decays each, from 7 patients, data is normalized to the peak intensity at ROI1 and presented as mean ± SD).

A total of 18 human cortical brain slices from seven patients were evaluated (Table 1). Slice viability was confirmed by electrical stimulation and induction of extracellular field potentials. An example is depicted in Fig. 1b (lower trace). Next, slices were incubated with iGlu_u_ (7 µl of an 8 µM solution) for 15 min using an interface-incubation loading technique^24^ (see methods) and transferred to a slice chamber at the microscope stage. The focus was set in the plane of the stimulation electrode (approx. 100 µm from the surface) using bright-field microscopy. To induce glutamate release, electrical stimulation (0.1 ms) was performed with the same intensity needed to induce a 50% of the maximum field potential response. Upon electrical stimulation in cortical layer V, we observed a steep intensity increase of the glutamate nanosensor fluorescence signal in the vicinity of the stimulation electrode. The intensity of the fluorescence signal subsequently decreased exponentially. A representative example of the glutamate nanosensor fluorescence signal dynamics as false-color image series as well as a plot of the intensity curve of the fluorescence signal is illustrated in Fig. 1a. Without stimulation, fluorescence intensity ΔF(t)/F_0_ showed a constant baseline around 0 with SD ± 0.03 in all patients. As depicted exemplarily in Fig. 1b, the dynamics of the glutamate nanosensor fluorescence signal (upper trace) showed a similar shape to that of the evoked extracellular field potentials (lower trace in Fig. 1b) as both signals were characterized by a strong marked increase followed by a slower decay. However, the fluorescence signal decayed much slower. Following electrical stimulation, the rapid transient increase in fluorescence intensity ΔF(t)/F_0_ peaked on average 545 ± 75 ms upon stimulation (n = 7 slices from 7 patients) The peak was on average 15 times higher than the standard deviation of the baseline signal.

**Table 1:** Patient characteristics. Abbreviations: F = female; M= male.

ID	Sex	Age [years]	Diagnosis	Hemisphere	Tissue	Number of slices
1	F	35	Hippocampal sclerosis	Right	Cortex	2
2	F	50	Hippocampal sclerosis	Left	Cortex	3
3	F	18	Hippocampal sclerosis	Left	Cortex	4
4	M	20	Hippocampal sclerosis	Right	Cortex	3
5	F	39	Hippocampal sclerosis	Left	Cortex	3
6	F	32	Encephalocele	Left	Cortex	2
7	M	59	Encephalocele	Left	Cortex	1

### Spatiotemporal properties of the nanosensor signal during glutamate transients.

We further investigated the spatial and temporal properties of the glutamate nanosensor fluorescence signal dynamics upon electrical stimulation. The signal progression was similar in all brain slices with a rapid steep rise followed by an exponential decay. Exponential fitting (R squared = 0.74) revealed a decay constant k = 0.36 as an indirect measure of the glutamate clearance from the ECS and a half-life of t_1/2_ = 1.95 s, as depicted in Fig. 1c (n = 7 slices from 7 patients, 63 decays).

In a next step, several regions of interest (ROIs) with increasing distance to the stimulation electrode in 100 µm steps were analyzed to investigate the spatial dependence of the iGlu_u_ fluorescence signal dynamics and consequently of glutamate concentration changes relative to the position of the stimulation electrode. Figure 1d is a schematic of 5 ROIs (yellow circles) chosen along an axis placed from the stimulation electrode perpendicular to the cortical surface with increasing distance to the electrode (black arrow). The diagram below visualizes the corresponding time courses for the iGlu_u_ fluorescence signal ratios (ΔF/F_0_), which revealed a clear decrease in the signal amplitudes with increasing distance to the electrode. An analysis of in total 56 time courses of the iGlu_u_ signal-ratio from 8 human brain slices from 7 patients at distances ranging from 0 to 400 µm in steps of 100 µm is depicted in Fig. 1e and showed a rapid decrease in fluorescence intensity change of 75% already at 100 µm away from the stimulation electrode.

**Figure 2:**
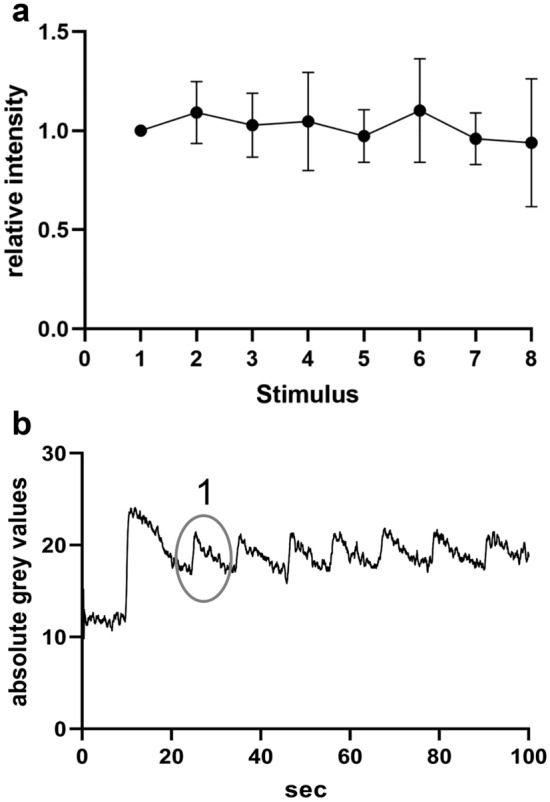
(**a**) Reproducibility of the iGlu_u_ fluorescence intensity change induced by bipolar stimulation. Peak iGlu_u_ fluorescent signal intensities in the proximity of the stimulation electrode for 8 repetitive stimulations with an interstimulus interval of 10 s (n = 7 slices from 7 patients, data is normalized to the first peak and presented as mean ± SD). **(b)** Example of a single experiment. Note the biggest peak after the first stimulation (excluded from analysis) followed by a change in baseline signal intensity superimposed by stable peaks upon further simulations. Circle indicates the first signal used for analysis and normalization -corresponding to the first value on the x axis in Fig. 2a.

Furthermore, we investigated the reproducibility of the experiment and found that the intensity of the iGlu_u_ fluorescent signal remained stable even after 8 repetitive stimulations every 10 s as the maximum signal intensities did not differ significantly between the stimulations (Fig. 2a, n = 7 slices/patients). An example trace is depicted in Fig. 2b. In individual experiments we tested the amount of photo-bleaching by comparing the F_0_ values preceding the second and last stimulus as described previously^23^. We observed no significant photo-bleaching, which was probably due to the short recording and exposure times.”

### Pharmacological manipulation of glutamate transience

**Figure 3:**
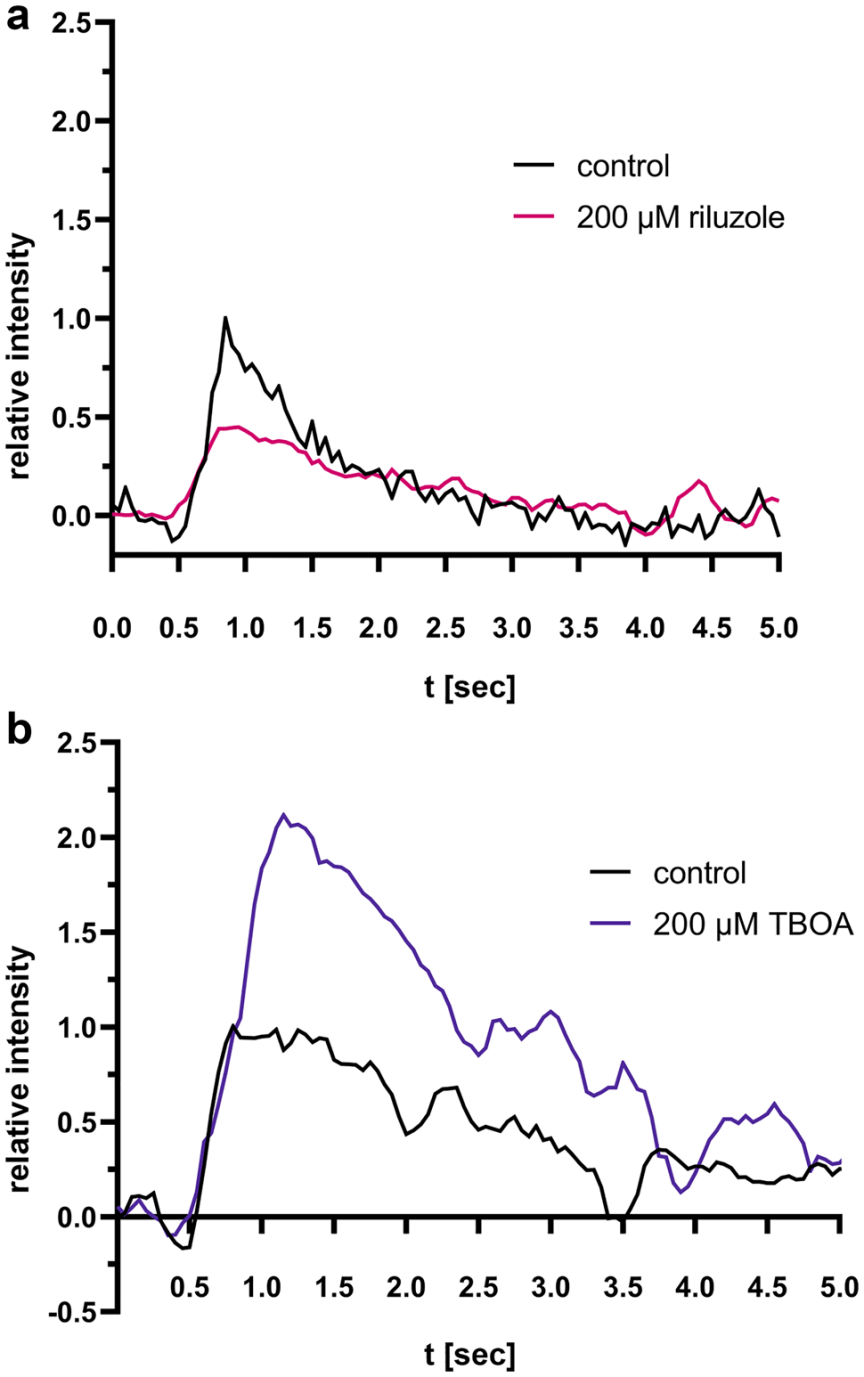
Glutamate nanosensor fluorescence dynamics can be manipulated by glutamate modulators. (**a**) The iGlu_u_ fluorescence response after application of the glutamate release inhibitor riluzole (7 µl of a 200 µM stock solution, applied in the vicinity of the stimulation electrode)—red trace—compared to the baseline measurement without pharmacological inhibition in the same slice (black trace). Traces represent averages of 16 stimulation cycles each (n = 2 slices from 2 patients), inter-stimulus interval 10 s. All values are normalized to the peak response of the baseline measurement. (**b**) The iGlu_u_ fluorescence signal intensity course after application of the glutamate reuptake inhibitor TBOA (7 µl of a 200 µM stock solution, applied in the vicinity of the stimulation electrode) -blue trace- compared to the baseline measurement without pharmacological inhibition (black trace). Traces represent averages of 16 stimulation cycles each (n = 2 slices from 2 patients), inter-stimulus interval 10 s. All values are normalized to the peak response of the baseline measurement.

Finally, we investigated the influence of pharmacological inhibition of the glutamate release or resorption on the iGlu_u_ fluorescence signal intensity. For this purpose, after obtaining baseline iGlu_u_ fluorescence responses, in a subset of slices a second measurement was performed upon two minutes incubation with a glutamate release modulator. Application of the glutamate release inhibitor riluzole^28^ (7 µl of a 200 µM stock solution, applied next to the stimulation electrode, n = 2 slices from 2 patients) prior to electric stimulation resulted in a distinct decrease in iGlu_u_ fluorescence signal intensity with a drop in peak intensity by 55% compared to the baseline measurement (Fig. 3a). By contrast, after application of the glutamate reuptake inhibitor TBOA (7 µl of a 200 µM stock solution), we detected a distinctly stronger rise in the iGlu_u_ fluorescence signal intensity with an increase in peak intensity by 112% compared to the baseline measurement (n = 2 slices from 2 patients, Fig. 3b).

## Discussion

In the present study, we successfully mapped the temporal and spatial glutamate dynamics in the extracellular space of acute human brain slices during physiologically tailored electrical stimulation using a single-FP-based intensiometric nanosensor. We adapted a protocol that was introduced for rat brain tissue by Dulla et al.^24^ for application at acute human cortical brain slices for the first time. By simply loading and incubating the slice with a medium containing a high concentration of the nanosensor, permeation throughout the ECS of the tissue was achieved. The electrically stimulated glutamate fluorescence nanosensor signals of the human cortical brain slices showed steep intensity increase followed by an exponential decrease. The spatial distribution and the time course of the signal were in good agreement with the position of the stimulation electrode and the dynamics of the electrical stimulation, respectively. Pharmacological manipulation of glutamate release and reuptake was associated with corresponding changes in the glutamate fluorescence nanosensor signals. Our findings were in good agreement with findings in previously published studies ^8,13,24,29,30^. Pharmacological blocking of glutamate release with riluzole led to a corresponding weakening of the fluorescence nanosensor response. Pharmacological inhibition of glutamate re-uptake, however, significantly increased the time to the peak glutamate level. These results confirm the feasibility and usability of this approach and demonstrate that intensiometric single-FP-based glutamate imaging shows great promise for high-resolution spatiotemporal imaging of glutamate transients and network activation in acute human brain slices.

The development of fluorescent biosensors was a milestone for investigating glutamatergic neurotransmission, allowing observation of excitatory synapses at work. However, studies of glutamate transients using FRET- or single-FP-based nanosensors have only been performed in animal models until now. The group of Prof. Dulla has pioneered the imaging of glutamate transients in the ECS using FRET-based nanosensors in rat brain slices and demonstrated that changes in network excitability and glutamate re-uptake alter evoked glutamate transients and produce correlated changes in evoked extracellular field potentials^24^. They concluded that the ability to perform real-time imaging of glutamate in slices should lead to key insights in brain function relevant to plasticity, development and pathology. In a follow-up study^31^ using this approach they showed in acutely disinhibited rat neocortical slices that robust release of glutamate during sustained epileptiform activity required neurons be provided a continuous source of glutamine and that the attenuation of network activity through inhibition of neuronal glutamine transport was associated with reduced frequency and amplitude of spontaneous events. In an animal model for cortical malformations induced by neonatal freeze-lesions they investigated alterations in glutamatergic signaling and found that asymmetric glutamate signals propagated throughout large areas of freeze-lesion cortex and the ability to remove exogenous glutamate was increased within the freeze-lesion itself but decreased in immediately adjacent regions with corresponding alterations in astrocyte density^25^. Gabapentin treatment immediately after freeze-lesion, however, prevented the formation of a hyperexcitable network and may help to minimize epileptogenic processes associated with developmental cortical malformations^32^. In a mouse model of traumatic brain injury they examined how cortical excitability and glutamatergic signaling was altered following injury and found that increased glutamatergic signaling due to loss of GABAergic control may provide a mechanism by which TBI can give rise to post-traumatic epilepsy^33^. Further studies using this simple nanosensor loading and incubating approach of rodent brain slices demonstrated that dynamic ambient glutamate signaling contributes to cortical interneuron maturation via tonic activation of GluN2C/GluN2D-containing NMDA receptors^34^ as well as that an astrocyte-dependent glutamate-glutamine cycle is required to maintain active neurotransmission at excitatory terminals^35^.

All these publications show the great potential of this method, but also the limitation that it has so far only been used in animal models and rodent cortical brain slices. Translation from animal models to human tissue has often met difficulties in the past. One reason is that human brain tissue is only available as a byproduct of neurosurgical resections for epilepsy or brain tumors which omits the possibility of proper “control” tissue. Besides, human tissue specimens show a tendency towards decreased excitability and require special conditions for eliciting network activity ^36,37^. Here, we report for the first time a feasible and simple protocol for real-time semi-quantitative glutamate imaging in an intact cortical brain slice with a temporal resolution in the millisecond range and a spatial resolution in the order of 50 µm range, which is similar to that of calcium imaging^38^ and capable of imaging glutamate responses to local electrical stimulation. The ability to use single-FP-based intensiometric nanosensors in live brain slices from patients is a next logical step forward in understanding pathophysiology of neurological disorders such as epilepsy.

There are several limitations of this approach. First, as with any imaging technique which is based on a binding-based detection method, the presence of a nanosensor protein may influence dynamics of endogenous glutamate transients by glutamate binding. The similar problem and possible ways of its resolution were recently described for calcium nanosensors^39^. Secondly, calibration of the glutamate FRET sensor has proven difficult due to the complex environment of brain tissue and influencing factors such as endogenous re-uptake or spontaneous release of glutamate. Furthermore, the fluorescence microscopy images in this study were collected with the surface plane of the slice in focus and, therefore, we only obtained fluorescent light from the surface of the brain slice although the nanosensor was present throughout the slice. Confocal and two-photon microscopy may allow a three-dimensional investigation and consequently a more precise spatial discrimination of neuronal structure. Finally, the use of the biosensors in the human brain is limited by the fact that normal brain tissue from healthy volunteers and consequently normal reference data are not available. We have circumvented this problem by using perilesional brain tissue, which was resected during epilepsy surgery. The integrity of the tissue cannot be guaranteed and physiological changes cannot be excluded. This might be the reason for the relatively long time period for glutamate clearance compared to data from animal models. Yet, since previous studies have used the less sensitive FRET sensor, it is possible that in our experiments much lower glutamate concentrations remaining in the ECS long after the electrochemical signal is abolished could be registered. Furthermore, a significantly higher glutamate concentration in the human ECS or impaired glutamate reuptake in the epileptic tissue ^40^ may contribute to the differences from experiments in rodent brain tissue.

Conclusively, we demonstrated that the recently developed fluorescent nanosensors for glutamate allow to detect neuronal activity in acute human cortical brain slices. Single-FP-based intensiometric nanosensors represent the most useful group of the nanosensors because they are monochromic and, thus, enable spectral multiplexing with other nanosensors or optogenetic tools, respectively. Future application to tissue samples from pathologies may provide new insights into pathophysiology without the limitations of an animal model. The application of fluorescent nanosensors for other neurotransmitters such as dopamine, acetylcholine, adrenaline, and GABA, possibly in combination with each other, using this approach also seem to be feasible.

## Methods

### Expression and purification of glutamate nanosensor

The glutamate nanosensor plasmid construct (pET41a iGlu_u_; a gift from Katalin Torok; addgene.org plasmid #119,834; http://n2t.net/addgene:119834) was transformed into BL21 (DE3) competent *E.coli* cells (New England Biolabs). Transformants were selected on LB plates enriched with kanamycin (50 µg/ml). After overnight incubation at 37 °C a single colony was inoculated in fresh 2xYT medium in presence of kanamycin (50 µg/ml) and shaken in the dark at 21 °C for one night. The expression of the His-tagged iGlu_u_ protein was induced by addition of 0.5 mM isopropyl β-D-1-thiogalactopyranoside (IPTG) into the 2xYT medium. Cells were collected by centrifugation at 4000 rpm (4 °C) for 40 min. Cell pellet was resuspended in extraction buffer consisting of 50 mM Na_3_PO_4_ and 300 mM NaCl, (pH 7.2), lysed with CelLytic™ B Cell Lysis Reagent (Sigma Aldrich), and dissolved by sonification on ice (Sonopuls, Bandelin). A cell-free extract was obtained by centrifugation at 12.000 rpm (4 °C) for 40 min and transferred to column containing HisPur™ Cobalt Resins (Thermoscientific) at 4 °C. The iGlu_u_ protein purification procedure was performed at 4 °C and included washing steps with extraction buffer and elution with extraction buffer containing 150 mM imidazole. Concentration of purified iGlu_u_ protein was determined spectroscopically (NanoVue Plus, Biochrom) and colorimetric using Pierce BCA Protein Assay (Thermoscientific). The fidelity of iGlu_u_ sensor was confirmed by sodium dodecyl sulphate–polyacrylamide gel electrophoresis (SDS-PAGE) and Coomassie staining. The aliquoted sensor was stored at -80 °C.

### Patients

Human brain tissue was obtained from seven patients (5 female, 2 male; mean age ± sd, 36.1 ± 14.9 years; age range 18–59 years) who underwent resective epilepsy surgery. All patients suffered from drug resistant temporal lobe epilepsy (TLE) and underwent a standardized anterotemporal en-bloc resection with or without transcortical amygdalohippocampectomy, depending on the presurgical assessment of the underlying focal epileptogenic lesion. A detailed overview of the patients` characteristics is presented in Table 1. All experiments were performed with brain tissue obtained from the temporal neocortex, which was not involved in the pathology and located outside the seizure focus. All studies were conducted in accordance with the Declaration of Helsinki and were approved by the local ethics committee of the Friedrich‐Alexander University Erlangen‐Nürnberg (FAU) Medical Faculty (Vote Nr. 193-18B). All patients gave informed consent prior to surgery.

### Slice preparation

A block of neocortex outside the structural epileptogenic lesion was transferred immediately after surgical removal to ice-cold artificial cerebrospinal fluid (aCSF; containing 129 mM NaCl, 3 mM KCl, 1.6 mM CaCl_2_, 1.8 mM MgSO_4_, 1.25 mM NaH_2_PO_4_, 21 mM NaHCO_3_, 10 mM glucose, saturated with carbogen: 95% O_2_, 5% CO_2_) in order to preserve vitality of the tissue during transfer to the lab. Overall transfer time did not exceed 10 min. The block of human neocortex was cut perpendicular to the pial surface into 400 µm thick slices using a vibratome (NT1000S, Leica) as described previously^41^. The human cortical brain slices were incubated at 32 °C in an interface-type recording chamber (Charité Berlin) perfused with carbogenated aCSF (flow rate: ∼1.8 ml/min, pH 7.4, osmolarity: 300 ± 3 mOsm/l). At least 60 min were allowed for slice equilibration.

### Field potential recordings for viability testing

Slice viability was tested by field potential recordings performed in the cortical layer II/III using chloride silver wire glass electrodes filled with aCSF (tip diameter 2 – 3 µm, resistance 2 to 4 MΩ). Signals were recorded with an extracellular differential amplifier (npi electronic), filtered at 3 kHz, and digitized with a sampling frequency of 10 kHz (Micro1401, Cambridge Electronic Design). Stimulation was performed with a bipolar tungsten electrode (Microprobes for Life Science; tip separation 75 µm, impedance 0.1 MΩ) via an isolated pulse stimulator (Model 2100, A-M Systems) within layer IV. Each pulse consisted of a 100 µs constant voltage stimulation. After initial input–output curves were generated, the baseline stimulation strength for the further experiments was adjusted to 40–60% of the maximum field excitatory potential response amplitude. Only slices with a response > 0.15 mV were used for the experiments with the glutamate nanosensors.

### Glutamate imaging of cortical human brain slices

For loading of the glutamate nanosensor protein into the ECS, the human cortical brain slice was transferred to a 35 mm cell imaging dish (Eppendorf) covered with carbogenated aCSF. The excess of aCSF was removed from the bottom of the dish as described previously and 7 µl of concentrated iGlu_u_ nanosensor solution (720 ng/µl corresponding to 8 µM) was carefully applied to the surface of the slice. The dish containing the slice was then placed in a humidified and warmed (37 °C) incubator (Heracell) equilibrated with 5% CO_2_. After 15 min of incubation, the slice was placed into the recording chamber mounted on a brightfield fluorescence microscope (Olympus IX71) equipped with a quick, high resolution software (CellF, Olympus) controlled CCD camera (F-View II, Olympus). Imaging was accomplished using a bandpass excitation (FWHM 470–490 nm) filter and emission (FWHM 510–550 nm) filter (Olympus, U-MNIBA2). Altogether 2401 images (344 × 258) were taken with a sampling frequency of 20 Hz (10 ms exposure time per image). Stimulation of slices with previously determined current intensity took place every 10 s.

Comparative measurements with the glutamate release inhibitor riluzole (Tocris Bioscience) and the glutamate reuptake inhibitor DL-threo-β-benzyloxyaspartic acid (TBOA; Tocris Bioscience) were performed on 2 slices each from 2 different patients. For this purpose, a standard measurement was first performed according to the above protocol. Afterwards, either 7 µl riluzole (200 µM) or 7 µl TBOA (200 µM) were applied directly to the same section, and separate glutamate imaging measurements using the stimulation protocol as described above were performed. The exposure time for both substances was 2 min. Drugs were applied using a local perfusion pipette for focal application. The dynamics of the glutamate nanosensor fluorescence intensity average over the same region of interest (ROI) without and during pharmacological inhibition were compared.

### Analysis of glutamate nanosensor data

Fluorescence microscopy data analysis of the glutamate nanosensor experiments was performed using the Fiji distribution of the open-source software package ImageJ^42^. Each fluorescence image stack was inspected visually in ImageJ for changes in fluorescence intensity after electrical stimulation in the vicinity of the stimulation electrode. Only slices showing peaks bigger than 3 × SD of the baseline noise were accepted for further analysis (success rate ~ 45% or 8 out of 18 slices). For analysis of iGluu temporal dynamics, in each slice a ROI with a diameter of 50 µm was manually set in the proximity (50 µm) of the stimulation electrode at an axis perpendicular to the cortical surface (See Fig. 1d, ROI 1). Background fluorescence (F_back_(t)) was measured from a remote ROI (diameter 50 µm) that did not show any intensity increase during the electrical stimulation. The fluorescence trace (F(t)) was corrected for background by subtracting F_back_ from the entire trace: ΔF(t) = F(t) − F_back_(t). For calculation of the relative fluorescence signal intensity (ΔF(t)/F_0_), F_0_ was first calculated from the average of a 500 ms window of points (10 images) prior to each stimulus and then subtracted from F(t) and used to scale the difference using the following equation:

$$\frac{\Delta F\left( t \right)}{{F_{0} }} = \frac{{F\left( t \right) - F_{0} }}{{F_{0} }}$$

Upon repetitive stimulation, the first stimulus always induced a much bigger increase in fluorescence intensity change than the following stimuli. This was probably due to unphysiologically strong release of glutamate from the readily releasable vesicle pool (RRP) by the electrical stimulation, which represents a much stronger and synchronized stimulus than physiologic activation by neuronal firing. By contrast, further stimuli caused smaller but stable fluorescence intensity changes. As transmitter release depends on a) RRP and b) vesicular release probability, these responses likely more accurately represented the glutamate release probability as the replenishment of the vesicle pool is expected to be constant at the constant time intervals^43^. The first stimulus was therefore excluded from analysis and for each slice averages of 7–9 consecutive stimuli with 10 s inter-stimulus interval were performed.

The procedure for fluorescence microscopy data analysis of glutamate imaging experiments using the iGlu_u_ nanosensor was described in detail previously^44^. Time courses of the glutamate nanosensor fluorescence intensity were plotted with Prism Graph Pad (version 9).

Statistical analyses were performed using SPSS (version 21, IBM). The temporal decay of the relative fluorescence signal intensity ΔF(t)/F_0_ was fitted by an exponential function:

$$\frac{\Delta F\left( t \right)}{{F_{0} }} = \frac{\Delta F\left( 0 \right)}{{F_{0} }} \times e^{ - kt}$$

ΔF(0) is the background corrected fluorescence trace ΔF(t) at t = 0 ms, and k is the decay constant that mathematically describes the fall of the iGlu_u_ fluorescence signal. Thus, k can be interpreted as an indirect measure for the extracellular glutamate clearance rate.

## References

[1] Cotman CW, Monaghan DT (1986). Anatomical organization of excitatory amino acid receptors and their properties. Adv. Exp. Med. Biol..

[2] Kandel ER (2001). The molecular biology of memory storage: A dialogue between genes and synapses. Science.

[3] Kirvell SL, Esiri M, Francis PT (2006). Down-regulation of vesicular glutamate transporters precedes cell loss and pathology in Alzheimer’s disease. J. Neurochem..

[4] Rothstein JD (1996). Excitotoxicity hypothesis. Neurology.

[5] Choi DW (1992). Excitotoxic cell death. J. Neurobiol..

[6] Belleroche JS, Bradford HF (1977). On the site of origin of transmitter amino acids released by depolarization of nerve terminals in vitro. J. Neurochem..

[7] Moore RY (1993). Principles of Synaptic Transmissiona. Ann. N. Y. Acad. Sci..

[8] Armbruster M, Hanson E, Dulla CG (2016). Glutamate clearance is locally modulated by presynaptic neuronal activity in the cerebral cortex. J. Neurosci..

[9] Auger C, Attwell D (2000). Fast removal of synaptic glutamate by postsynaptic transporters. Neuron.

[10] Diamond JS (2005). Deriving the Glutamate clearance time course from transporter currents in CA1 Hippocampal Astrocytes: Transmitter uptake gets faster during development. J. Neurosci..

[11] Barbour B, Häusser M (1997). Intersynaptic diffusion of neurotransmitter. Trends Neurosci..

[12] Rusakov DA, Kullmann DM (1998). Extrasynaptic Glutamate diffusion in the hippocampus: Ultrastructural constraints, uptake, and receptor activation. J. Neurosci..

[13] Okubo Y (2010). Imaging extrasynaptic glutamate dynamics in the brain. Proc. Natl. Acad. Sci..

[14] Zhang S (2005). Measurement of GABA and glutamate in vivo levels with high sensitivity and frequency. Brain Res. Protoc..

[15] Benveniste H, Drejer J, Schousboe A, Diemer NH (1984). Elevation of the extracellular concentrations of glutamate and aspartate in rat hippocampus during transient Cerebral Ischemia monitored by intracerebral microdialysis. J. Neurochem..

[16] Innocenti B, Parpura V, Haydon PG (2000). Imaging extracellular waves of glutamate during calcium signaling in cultured astrocytes. J. Neurosci..

[17] Nicholls DG, Sihra TS (1986). Synaptosomes possess an exocytotic pool of glutamate. Nature.

[18] Oldenziel WH, Dijkstra G, Cremers TIFH, Westerink BHC (2006). In vivo monitoring of extracellular glutamate in the brain with a microsensor. Brain Res..

[19] Hu Y, Mitchell KM, Albahadily FN, Michaelis EK, Wilson GS (1994). Direct measurement of glutamate release in the brain using a dual enzyme-based electrochemical sensor. Brain Res..

[20] Förster T (1948). Zwischenmolekulare Energiewanderung und Fluoreszenz (Intermolecular energy migration and fluorescence). Ann. Phys..

[21] Okumoto S (2005). Detection of glutamate release from neurons by genetically encoded surface-displayed FRET nanosensors. Proc. Natl. Acad. Sci. USA.

[22] Hires SA, Zhu Y, Tsien RY (2008). Optical measurement of synaptic glutamate spillover and reuptake by linker optimized glutamate-sensitive fluorescent reporters. Proc. Natl. Acad. Sci. USA.

[23] Helassa N (2018). Ultrafast glutamate sensors resolve high-frequency release at Schaffer collateral synapses. Proc. Natl. Acad. Sci. USA.

[24] Dulla C (2008). Imaging of glutamate in brain slices using FRET sensors. J. Neurosci. Methods.

[25] Dulla CG, Tani H, Brill J, Reimer RJ, Huguenard JR (2013). Glutamate biosensor imaging reveals dysregulation of glutamatergic pathways in a model of developmental cortical malformation. Neurobiol. Dis..

[26] Lamas JA, Selyanko AA, Brown DA (1997). Effects of a Cognition-enhancer, Linopirdine (DuP 996), on M-type Potassium Currents (I K(M) ) some other voltage- and ligand-gated membrane currents in rat sympathetic neurons. Eur. J. Neurosci..

[27] Maslarova A (2011). Chronically epileptic human and rat neocortex display a similar resistance against spreading depolarization in vitro. Stroke.

[28] Doble A (1996). The pharmacology and mechanism of action of riluzole. Neurology.

[29] Parsons MP (2016). Real-time imaging of glutamate clearance reveals normal striatal uptake in Huntington disease mouse models. Nat. Commun..

[30] Pinky NF, Wilkie CM, Barnes JR, Parsons MP (2018). Region- and activity-dependent regulation of extracellular glutamate. J. Neurosci..

[31] Tani H, Dulla CG, Huguenard JR, Reimer RJ (2010). Glutamine Is required for persistent epileptiform activity in the disinhibited neocortical brain slice. J. Neurosci..

[32] Andresen L (2014). Gabapentin attenuates hyperexcitability in the freeze-lesion model of developmental cortical malformation. Neurobiol. Dis..

[33] Cantu D (2015). Traumatic brain injury increases cortical glutamate network activity by compromising GABAergic control. Cereb. Cortex.

[34] Hanson E (2019). Tonic activation of GluN2C/GluN2D-containing NMDA receptors by ambient glutamate facilitates cortical interneuron maturation. J. Neurosci..

[35] Tani H (2014). A local glutamate-glutamine cycle sustains synaptic excitatory transmitter release. Neuron.

[36] Pitkänen, A., Buckmaster, P. S., Galanopoulou, A. S. & Moshé, S. L. *Models of Seizures and Epilepsy: Second Edition*. *Models of Seizures and Epilepsy: Second Edition* (2017).

[37] de la Prida LM, Huberfeld G (2019). Inhibition and oscillations in the human brain tissue in vitro. Neurobiol. Dis..

[38] Russell JT (2011). Imaging calcium signals in vivo: A powerful tool in physiology and pharmacology. Br. J. Pharmacol..

[39] McMahon SM, Jackson MB (2018). An inconvenient truth: Calcium sensors are calcium buffers. Trends Neurosci..

[40] Heinemann U, Kaufer D, Friedman A (2012). Blood-brain barrier dysfunction, TGFβ signaling, and astrocyte dysfunction in epilepsy. Glia.

[41] Kann O (2005). Metabolic dysfunction during neuronal activation in the ex vivo hippocampus from chronic epileptic rats and humans. Brain.

[42] Schindelin J (2012). Fiji: An open-source platform for biological-image analysis. Nat. Methods.

[43] Kaeser PS, Regehr WG (2017). The readily releasable pool of synaptic vesicles. Curr. Opin. Neurobiol..

[44] Marvin JS (2018). Stability, affinity, and chromatic variants of the glutamate sensor iGluSnFR. Nat. Methods.

